# Editorial Notes

**Published:** 1882-10

**Authors:** 


### EDITORIAL NOTES.

LACTOPEPTINE.—We call attention to the advertisement of the New York Pharmacal Association, and particularly to the certificate of Prof. Attfeild, of London, as to the composition and properties of *Lactopeptine*. It has for years been a favorite remedy of ours, and we are glad to learn that it is being very largely used in England.

THE August number of the *Edinburgh Medical Journal* has the following handsome notice of the preparations of a well-known New York firm. As an evidence of foreign appreciation of American skill, and as an echo from across the sea of what we have ourselves said of the preparations of this old and trusty house, we quote the notice entire:

"W. H. SCHIEFFELIN & CO.'S SOLUBLE PILLS AND GRANULES (New York).—We have been favored with a case containing one dozen bottles of these pills and granules, and we have no hesitation in highly commending them to the notice of the profession. The pills are perfectly globular, and finished with a beautiful nicety surpassing anything we have before witnessed. They are covered with a thin, transparent coating, which is perfectly tasteless and easily soluble in the mouth or stomach. This coating is so thin that it does not add materially to the bulk of the pill, and, being transparent, discloses to the eye the exact color and appearance of the pill-masses, and thus tends, in no small degree, to prevent one pill being mistaken for another. This coating appears to be quite sufficient to protect the pills from atmospheric influences, and to preserve the soluble condition of the mass. All pills should be sufficiently consistent to maintain their globular form, and yet sufficiently soft to melt in the stomach. These two conditions are possessed in a remarkable degree by Schieffelin's pills and granules. Another excellent property possessed by many of these pills is their small size, many of them being *very small*, such as those containing morphia, digitalin, etc. They prepare a pill containing 'ergotin,' their 3-grain pill representing 15 to 17 grains of ergot. This pill is a valuable addition to our armamentarium. The firm of W. H. Schieffelin & Co., of New York, holds the highest place among American pharmaceutical chemists, and consequently the quality and quantity of the various ingredients can be perfectly relied on. The price of the pills is very moderate, and quite within the reach of the great majority of patients. The case was accompanied with a 'Formula List, with Notes,' giving the composition of each pill and the price. For perfection of finish these pills excel any pills we have ever seen, and we believe they require only to be known to the profession to be extensively prescribed."

The world-wide reputation of the *Edinburgh Medical Journal* makes such a notice of great value.

